# Effect of combined dexmedetomidine with ropivacaine in transversus thoracis plane block on surgical stress response during cardiopulmonary bypass surgery: a randomized controlled trial

**DOI:** 10.3389/fcvm.2025.1638373

**Published:** 2025-10-10

**Authors:** Lian Huang, Ying Chen, Xiaohua Xu, Fanpei Zeng, Chuntian Li, Hangxiang Fu

**Affiliations:** ^1^ Department of Anaesthesiology, Longyan First Affiliated Hospital of Fujian Medical University, Longyan, China; ^2^ Department of Laboratory Medicine, Longyan First Affiliated Hospital of Fujian Medical University, Longyan, China

**Keywords:** transversus thoracis muscle plane block, open cardiac surgery, dexmedetomidine, stress response, postoperative pain

## Abstract

**Objective:**

To examine how patients having open heart surgery under cardiopulmonary bypass (CPB) react to surgical stress following bilateral transversus thoracis plane (TTP) block with ropivacaine improved by dexmedetomidine (DEX).

**Methods:**

Three groups of sixty patients (26M/34F, ASA II–III, 18–65 years old) slated for elective CPB heart surgery were randomly assigned: general anesthesia alone (Group C), TTP (ropivacaine) combined with general anesthesia group (Group R), or TTP (ropivacaine + DEX) combined with general anesthesia group (Group RD). Primary outcomes measured serum cortisol levels at five perioperative phases, while the secondary outcomes included glucose/C-reactive protein (CRP) levels, Numeric Rating Scale (NRS) pain scores postextubation, 48-hr sufentanil consumption, patient-controlled analgesia (PCA) demand frequency, rescue analgesia rates, mechanical ventilation duration, ICU stay, and complications.

**Results:**

At 24 h postoperatively, RD and R groups exhibited statistical lower serum cortisol levels compared to controls (*p* < 0.05), with parallel glucose reductions. However, the CRP level increased significantly. NRS scores in RD/R groups were significantly lower than controls at 0 h, 6 h, and 12 h postextubation (*p* < 0.05), and the RD group maintained superior analgesia vs. both groups at 24 h. RD and R groups demonstrated significant reductions for 48-h sufentanil consumption vs. controls, and RD group showed less total sufentanil consumption vs. R group. Besides, both mechanical ventilation duration and ICU stay were shortened by serval hours compared to control. Significant reductions in the count of effective analgesic pump compressions were observed in groups R and RD compared to the control group. Moreover, rescue analgesia rates were 55%, and 15% lower in RD vs. R and Control groups, respectively (*p* = 0.031). However, no intergroup differences occurred pulmonary complications.

**Conclusion:**

DEX-enhanced TTP blockade may contribute to attenuating the stress response, optimizing analgesia, and improving early postoperative recovery parameters in CPB cardiac surgery through opioid-sparing mechanisms and sympatholytic effects, demonstrating clinical viability within Enhanced Recovery After Surgery (ERAS) protocols.

**Clinical Trial Registration:**

https://www.chictr.org.cn/index.html, identifier ChiCTR2400085899.

## Introduction

Conventional general anesthesia during cardiopulmonary bypass (CPB)-assisted open-heart surgery presents notable limitations. First, pharmacologically induced cardiovascular depression often precipitates hemodynamic instability characterized by hypotension and arrhythmias (1, 2). Second, suboptimal myocardial protective effects may exacerbate ischemia-reperfusion injury, correlating with impaired postoperative ventricular function (3, 4). Third, the systemic inflammatory response caused by this approach increases the incidence of lung-related issues, including acute respiratory distress syndrome (ARDS) (5). Therefore, anesthesia management during CPB requires precise drug adjustment and monitoring to balance the anesthetic depth and hemodynamic stability.

Combining regional anesthesia (RA) with general anesthesia (GA) in open heart surgery provides several significant advantages (6), such as reducing oxidative stress, improving pulmonary complications and postoperative pain, and decreasing the reliance on opioids (7). The transversus thoracis plane block, a form of peripheral thoracic nerve block, proficiently shuts down the anterior divisions of the T2 through T6 intercostal nerves, thereby delivering pain relief for surgeries on the anterior chest wall (8, 9). This approach has been suggested for cardiac procedures in certain studies, demonstrating both effectiveness and safety (10). Long-acting local anesthetics, like ropivacaine and bupivacaine, can certainly enhance pain relief post-surgery (11, 12). Nonetheless, the sensory numbness they provide is still too short to eliminate the need for opioids entirely after an operation, and their overall effectiveness is questionable (13). Consequently, increasing the longevity of nerve blocks to manage postoperative discomfort is a crucial challenge in the field of regional anesthesia.

Dexmedetomidine, a potent and selective α2-adrenergic receptor agonist, is known for its multifaceted pharmacological properties, including sedation, pain relief, anti-inflammatory action, and suppression of sympathetic activity (14). It has been recognized as a safe and effective adjunct in various anesthetic applications and analgesic techniques (15). Its incorporation with local anesthetics has been shown to extend regional anesthesia, speed up the onset of numbness, improve postoperative pain relief, and reduce the need for additional pain medications (16, 17). In this study, we combined dexmedetomidine (DEX) and ropivacaine in bilateral TTP block regimens to observe the effects on stress response of open-heart surgery under CPB, and our findings preliminarily confirmed these clinical benefits.

## Methods

This prospective randomized controlled trial, conducted exclusively at The First Affiliated Hospital of Fujian Medical University in Longyan, received ethical clearance from the institutional review board after securing written consent from every participant. Prior to commencement, the research protocol was officially registered with the Chinese Clinical Trial Registry under the identifier ChiCTR2400085899.

### Subjects

Eligible participants were adults aged 18–65 years with ASA physical status II–III scheduled for open heart surgery under CPB. Exclusion criteria comprised (i) refusal to receive transverse thoracic muscle plane block; (ii) hypersensitivity to local anesthetics or opioids; or (iii) preexisting hepatic/renal insufficiency, cardiac dysfunction, reoperation, coagulation dysfunction, and communication barriers.

### Randomization and blinding

Using computer-generated randomization with permuted blocks (1:1:1 allocation), participants were allocated to three groups: standard general anesthesia (C Group), ropivacaine-assisted general anesthesia (Ropivacaine Group), or DEX-enhanced ropivacaine general anesthesia (RD Group). The sealed envelopes containing group assignments were managed by an independent statistician, and the intervention solutions were prepared by a non-participant anesthesia nurse. The injection standards for each group are as follows: The C group received general anesthesia alone; the R group received an injection of 0.3% or 0.5% ropivacaine in the transverse thoracic muscle plane; and the RD group received an injection of 0.3% or 0.5% ropivacaine combined with dexmedetomidine in the transverse thoracic muscle plane. As TTP block surgery is an operation guided by ultrasound and requires a high level of technical proficiency, it can be performed by experienced anesthesiologists without blinding. However, to minimize bias, all postoperative assessors, ICU management teams, and statistical analysts were unaware of the grouping situation.

### Anaesthesia

Standardized monitoring including pulse oximetry, five-lead electrocardiography, and non-invasive blood pressure was established prior to administering 5 μg sufentanil IV. Radial artery cannulation under local anesthesia enabled continuous invasive arterial pressure monitoring. The anesthesia plan began with a series of medications: midazolam (given in amounts ranging from 0.05 to 0.1 mg per kilogram of body weight), sufentanil (0.4–0.8 μg per kilogram), propofol (2–2.5 mg per kilogram), and rocuronium (0.9 mg per kilogram), and concluded with the insertion of a breathing tube and the start of mechanical ventilation. Ultrasound-guided right internal jugular venous catheterization facilitated central venous pressure monitoring.

Intervention groups received bilateral ultrasound-guided transversus thoracis plane blocks with ropivacaine (R group) or ropivacaine plus dexmedetomidine (RD group), while controls received no treats. Throughout the surgical procedure, anesthesia was maintained using a combination of sevoflurane (0.8–1 MAC), propofol (4–12 mg/kg/h), and remifentanil (0.025–2 μg/kg/min), supplemented by intermittent IV boluses of rocuronium (0.1 mg/kg). The bispectral index was carefully adjusted and kept within the target range of 40–60 for the duration of the operation.

Hemodynamic support medications were administered per institutional protocols. Before the end of surgery, preemptive analgesia with flurbiprofen axetil 50 mg was administered, along with ondansetron 4 mg IV for antiemetic prophylaxis. All procedures were performed by a designated cardiothoracic surgical team, followed by protocolized transfer to the cardiac surgical intensive care unit.

Postoperative analgesia included: (i) PCIA pump containing sufentanil 100 μg and ondansetron 8 mg in 100 ml saline (basal 1 ml/h, bolus 3 ml, 20-min lockout); (ii) scheduled flurbiprofen axetil 50 mg IV q8h. Rescue morphine 0.1 mg/kg IV was administered for numeric rating scale (NRS) ≥4 despite maximal PCA use.

### Ultrasound-guided transversus thoracis muscle plane block

Ultrasound-guided TTP blocks were performed under strict aseptic technique using a high-frequency linear probe. Patients were positioned supine with bilateral access at the 4th-5th intercostal parasternal regions. Sonographic identification of anatomical landmarks included visualization of the pectoralis major, external/internal intercostal muscles, internal thoracic vasculature, transversus thoracis muscle, and pleural interface. The internal thoracic artery/vein complex served as key anatomical landmarks for target plane localization. Employing an in-plane technique, a 20 G puncture needle was guided into the surface of the transversus thoracis muscle under the real-time ultrasound observation. Successful block was confirmed as the significantly depressed of pleura (18). Ropivacaine dosing was stratified by body weight: 20 ml 0.3% (<75 kg) or 0.5% (≥75 kg) per hemithorax (6). The RD group received adjunctive dexmedetomidine 1 μg/kg mixed with ropivacaine. All blocks were performed by a single regional anesthesia specialist with extensive experience in ultrasound-guided regional anesthesia (19).

### Outcomes

The primary outcome measured serum cortisol levels at five defined perioperative phases: preoperative baseline upon operating room entry (T0), during sternotomy (T1), at cardiopulmonary bypass termination (T2), upon surgical completion (T3), and 24 h postoperatively (T4). Secondary outcomes encompassed serial measurements of serum glucose and C-reactive protein (CRP) at matching time points, pain intensity via Numeric Rating Scale (NRS 0–10) assessed immediately postextubation and at 6, 12, 24, and 48 h thereafter, cumulative 48-h sufentanil consumption with patient-controlled analgesia (PCA) demand frequency, rescue analgesia requirements, mechanical ventilation duration, ICU length of stay, incidence of pulmonary complications (pneumonia/pleural effusion), and postoperative nausea/vomiting events. All blood samples were taken from deep veins and sent for testing promptly after collection.

### Statistical analysis

This single-center exploratory feasibility study was designed to evaluate the clinical feasibility and safety of DEX combined with TTP block in CPB surgery. As there was no prior data available in this field, sample size estimation was not performed. The sample size of 60 patients (20 per arm) was chosen to provide descriptive estimates rather than definitive hypothesis testing. Consequently, all secondary analyses are exploratory and hypothesis-generating only. All statistical analyses were performed using SPSS version 22.0 and R version 4.22. For continuous variables, the Shapiro–Wilk test was used to assess normality, supported by visual inspection via Q-Q plots and histograms. Normally distributed data were presented as mean ± standard deviation, while skewed data were described using medians and interquartile ranges. Between-group comparisons were carried out using one-way ANOVA for normally distributed data and the Kruskal–Wallis test followed by Dunn's *post hoc* test for non-parametric data. Repeated-measures data were analyzed using linear mixed-effects models to account for within-subject correlations. Categorical variables were summarized as percentages and analyzed with chi-square tests or Fisher's exact tests, depending on the sample size and distribution. Statistical significance was set at *p* < 0.05 for all tests.

## Results

**Figure 1:**
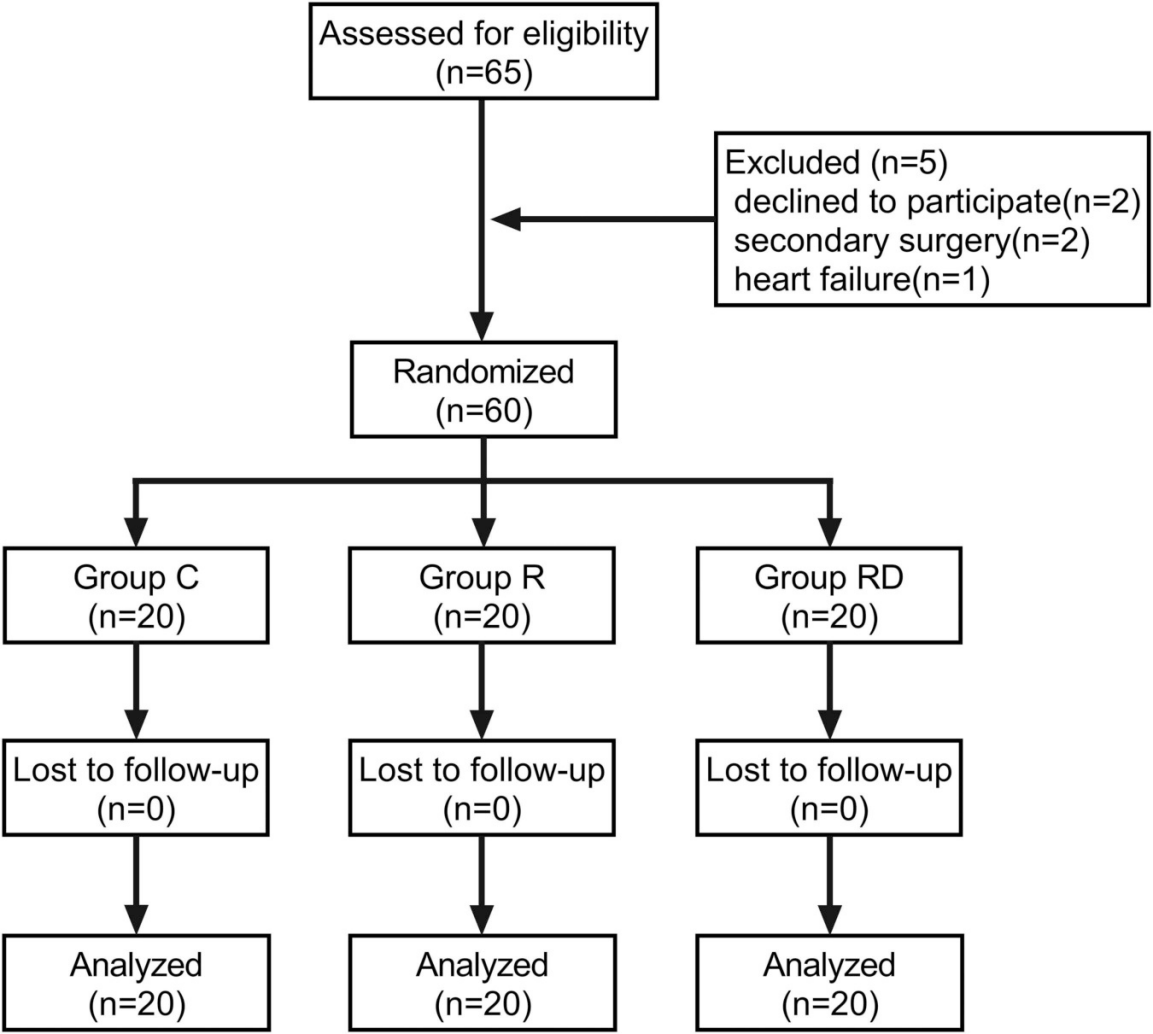
Study design and recruitment outline. Study flow diagram showing the number of subjects screened, enrolled, randomized and included in the primary analysis.

Between October 2022 and July 2024, 65 consecutive patients were screened for eligibility. Five participants were excluded (two declined participation, two required re-operative cardiac procedures, one developed decompensated heart failure), resulting in 60 patients (20 per group) completing the study protocol and included in the final analysis cohort (Figure 1). Baseline demographic and perioperative data (age, sex, BMI, ASA status, LVEF, intraoperative time, CPB time) are presented in Table 1, demonstrating comparable characteristics across groups (*p* > 0.05).

**Table 1:** Demographic and perioperative characteristics.

Clinical variables	Group C (*n* = 20)	Group R (*n* = 20)	Group RD (*n* = 20)	*P*-value
Gender (male/female)	11/9	8/12	7/13	0.414
Age (year)	56.00 (10.50)	55.50 (14.75)	52.00 (12.25)	0.883
BMI (kg/m^2^)	23.07 ± 3.16	24.34 ± 3.75	23.06 ± 2.75	0.363
ASA (Ⅱ/Ⅲ)	8/12	7/13	6/14	0.803
LVEF (%)	61.55 ± 4.00	61.00 ± 6.37	60.65 ± 4.80	0.857
Total intraoperative time (min)	230.65 ± 36.26	217.00 ± 40.08	226.50 ± 39.21	0.521
Cardiopulmonary bypass time (min)	92.50 (32.50)	95.00 (22.33)	107.50 (37.50)	0.300

Numbers in brackets indicate patients included in each group. BMI, body mass index.

**Figure 2:**
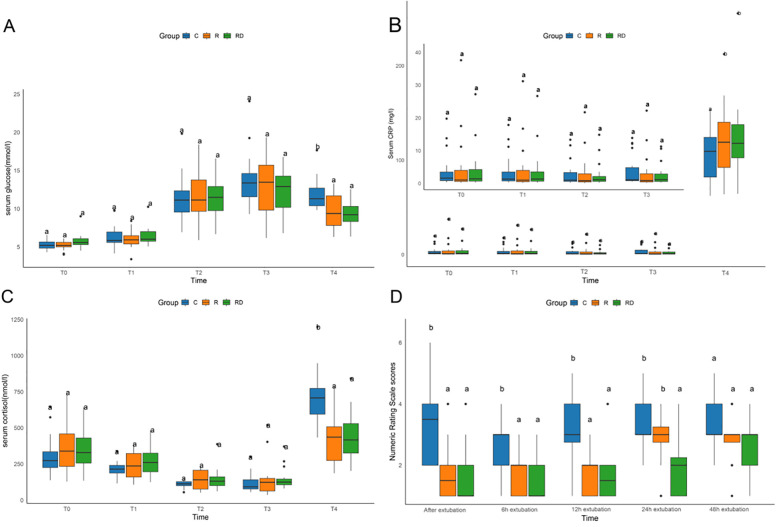
Comparison of serum glucose, CRP, cortisol, and numeric rating scale among C, R and RD groups. **(A–C)** Comparison of serum glucose, CRP, and cortisol among three groups at five defined perioperative phases. **(D)** Comparison of Numeric Rating Scale among three groups at distinct postextubation time point. T0, preoperative baseline upon operating room entry; T1, during sternotomy; T2, at cardiopulmonary bypass termination; T3, upon surgical completion; T4, 24 h postoperatively. Lowercase letters (a, b) denote statistically significant differences compared to the control group **(C)** at each time point (Dunnett's *post hoc* test, *p* < 0.05).

As depicted in Figure 2, 24 h post-surgery, both the R and RD groups exhibited significantly reduced serum cortisol levels and blood glucose concentrations compared to the control group, accompanied by a significant elevation in CRP levels (*p* < 0.05). However, no significant differences were observed between the R and RD groups themselves. Postoperative pain assessment demonstrated superior analgesic efficacy in the intervention groups, with R and RD cohorts showing significantly lower NRS scores vs. controls at extubation (0 h), 6 h, and 12 h post-extubation (all *p* < 0.05). By 24 h, RD maintained analgesic superiority over both control and R groups, while R group scores converged with controls. All intergroup NRS differences resolved by 48 h postextubation (*p* > 0.05), as detailed in Figure 2.

**Figure 3:**
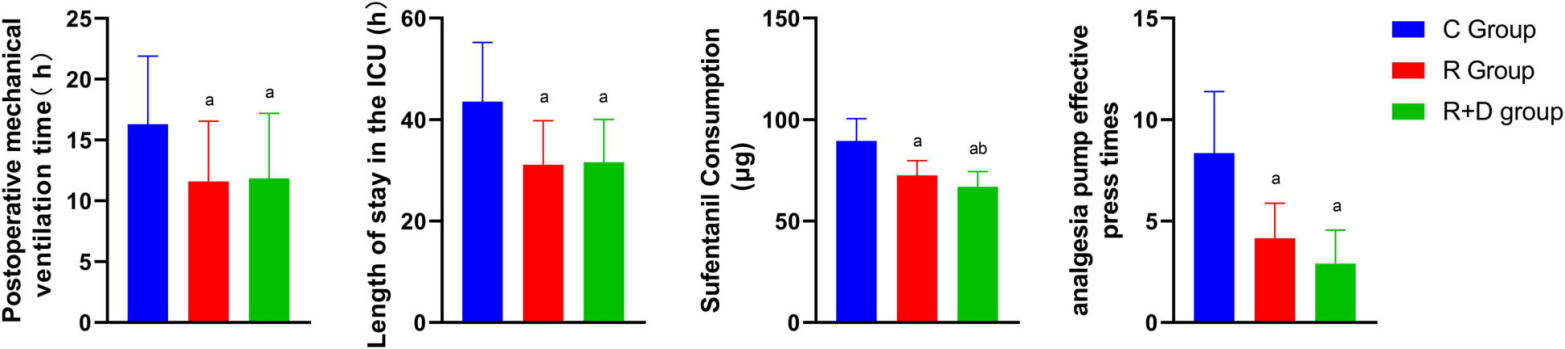
Comparison of mechanical ventilation duration, ICU length of stay, sufentanil consumption, and analgesia requirements among three groups. Blue represents group C (Control), red represents group R (Ropivacaine), and green represents group RD (DEX-enhanced ropivacaine). *p* ^a^ <0.05 versus Group C, *p* ^b^ <0.05 versus group R.

The RD group demonstrated significantly shorter postoperative mechanical ventilation duration and ICU length of stay compared to controls (*p* < 0.05). Postextubation analgesic requirements were markedly reduced in both intervention groups, with R and RD cohorts showing 48-h cumulative sufentanil consumption lower than controls, and the RD group also used significantly less sufentanil than the R group. Moreover, the demand frequency of PCA followed similar patterns, with intervention groups exhibiting fewer effective bolus requests vs. controls as illustrated in Figure 3.

Other postoperative outcomes contained the incidence of postoperative nausea, vomiting, pulmonary complications, and postoperative rescue rate, which were shown in Table 2. No intergroup differences emerged in PONV (15%–40% overall, *p* = 0.155) or pulmonary complication rates (25%–30% incidence, *p* = 0.921). However, the RD group demonstrated superior analgesic sustainability, requiring 55% and 15% fewer rescue interventions than controls and R group respectively (*p* < 0.001).

**Table 2:** Postoperative clinical outcomes.

Incidence of adverse events	Group C (*n* = 20)	Group R (*n* = 20)	Group RD (*n* = 20)	*P*-value
Incidence of PONV (%)	8 (40.0%)	4 (20%)	3 (15%)	0.155
Incidence of pulmonary complication (%)	6 (30%)	5 (25%)	6 (30%)	0.921
Incidence of rescue analgesia (%)	14 (70%)	6 (30%)	3 (15%)	<0.001

Numbers in brackets indicate patients included in each group. PONV, postoperative nausea and vomiting.

## Discussion

This randomized controlled trial establishes that dexmedetomidine-enhanced TTP blockade significantly improved postoperative outcomes in cardiac surgery patients undergoing CPB. The intervention demonstrated potential tripartite benefits: (i) superior analgesia extending through 24 h postextubation; (ii) attenuation of surgical stress biomarkers; and (iii) accelerated functional recovery as evidenced by reduced mechanical ventilation duration and ICU stay. These results underscored the significant potential of dexmedetomidine as a valuable adjunct in regional anesthesia for complex surgical procedures, thereby enhancing the overall efficacy and safety of such interventions. Notably, the ropivacaine concentration selection (0.3% for patients <70 kg, 0.5% for ≥70 kg) was based on weight-dependent pharmacokinetics, ensuring adequate blockade while minimizing potential toxicity in smaller patients (20, 21). Although it was expected that the CRP levels in R and RD groups would be lower than control group, the results showed an increase. This might be due to the small sample size. With only about 20 patients per group, there may be insufficient statistical power, increasing the risk of type II errors and masking actual differences. In addition, small samples are more susceptible to random fluctuations, leading to results that deviate from expectations.

The observed reduction in 48-h sufentanil consumption in the RD group, coupled with significant lower rescue analgesia requirements compared to controls, underscored dexmedetomidine's multimodal action. Beyond its recognized α2-adrenergic mediated sympatholytic effects, our biomarker data suggest peripheral modulation of surgical stress pathways. The sustained cortisol suppression at 24 h postoperatively correlated with dexmedetomidine's known inhibition of hypothalamic corticotropin-releasing hormone secretion (22), while attenuated hyperglycemia likely reflects improved insulin sensitivity through reduced catecholamine surge (23).

Clinically, certain important recovery metrics demonstrated the benefit brought from optimized analgesia. The RD group exhibited shorter mechanical ventilation duration and reduced ICU stay, which likely stem from enhanced diaphragmatic function preservation. These outcomes were in line with recent thoracic surgical trials demonstrating that regional anesthesia adjuncts reduce pulmonary complications (23). Besides, NRS were consistently lower in the RD group, particularly during the first 24 h following extubation, demonstrating the ability of dexmedetomidine to prolong sensory blockade when combined with local anesthetics (24). Notably, the opioid-sparing effect persisted even when compared to the R group, indicating dexmedetomidine as an independent role in pain management. These findings hold substantial clinical significance, as opioid-related adverse effects, including respiratory depression, ileus, and delirium, continue to be primary concerns for patients undergoing cardiac surgery (25). Based on TTP block, the combination of dexmedetomidine has solved the problem of relatively limited sustainable duration of analgesia (26). Dexmedetomidine has been proven to prolong the duration of peripheral nerve block by inhibiting the hyperpolarization-activated cation current and enhancing the binding of local anesthetics to sodium channels. Similar results have also been reported in other local techniques, such as interscalene block and femoral nerve block (27). Although the plasma half-life of dexmedetomidine is approximately 2 h, this study found that its analgesic effect lasts more than 24 h. This might be due to its high lipophilicity, which enables it to form tissue reservoir and continuous release, as well as its direct effect on the peripheral α2-adrenergic receptors of the dorsal horn, thereby enhancing and prolonging the effect of local anesthetics (28, 29).

The observed decrease in stress biomarkers (cortisol, glucose) aligns with findings from trials assessing dexmedetomidine in major abdominal surgeries (30). Importantly, the drug's anti-sympathetic effects may also help stabilize CPB-induced hemodynamic fluctuations, possibly diminishing the need for vasopressors or inotropes (31). Nevertheless, this study did not specifically evaluate catecholamine levels or the requirements for vasoactive drugs, thus highlighting a need for further research. Notably, the absence of significant differences in pulmonary complications or PONV across groups was surprising. The universal ondansetron administration may have masked dexmedetomidine's antiemetic properties, potentially underestimating its PONV reduction potential.

This study has several limitations. Firstly, the lack of a pre-trial power calculation and the modest sample size may limit the reliability of subgroup comparisons and time-based interaction analyses. Future studies should consider adaptive or sequential designs to enhance statistical efficiency. Secondly, potential performance bias may have arisen from the unblinding of anesthesiologists performing the TTP blocks, which could have influenced intraoperative management and dosing decisions. The exclusion of high-risk or re-operative patients also restricts the generalizability of our findings. Thirdly, other clinically relevant outcomes were not assessed. We acknowledge that this trial did not evaluate long-term endpoints, such as chronic postoperative pain, persistent opioid use, or late complications. Future studies should include extended follow-up and broader outcome measures. Fourthly, lack of serial plasma catecholamine assays limits the mechanistic interpretation of dexmedetomidine's α2-mediated effects during CPB. Lastly, the use of different ropivacaine concentrations stratified by body weight may have introduced unmeasured pharmacokinetic differences linked to body composition.

In summary, this randomized controlled trial suggests that adding dexmedetomidine to ropivacaine for the TTP block may help attenuate the stress response, optimise analgesia, and improve early postoperative recovery parameters in cardiac surgery with cardiopulmonary bypass. These advantages are consistent with ERAS goals and highlight the potential of adjunctive α2-agonists in regional anesthesia. Although limitations exist, the results provide a strong basis for integrating dexmedetomidine-enhanced TTP blocks into cardiac surgical practice, potentially transforming perioperative care paradigms. Further multi-center trials with larger sample sizes are necessary to confirm these findings and investigate subgroup analyses, such as in elderly patients or those with diabetes.

## Data Availability

The datasets presented in this study can be found in online repositories. The names of the repository/repositories and accession number(s) can be found in the article/Supplementary Material.
